# Clinical Characteristics and Management of 50 Patients with Anti-GAD Ataxia: Gluten-Free Diet Has a Major Impact

**DOI:** 10.1007/s12311-020-01203-w

**Published:** 2020-10-21

**Authors:** M. Hadjivassiliou, P. G. Sarrigiannis, P. D. Shanmugarajah, D. S. Sanders, R. A. Grünewald, P. Zis, N. Hoggard

**Affiliations:** 1 Academic Department of Neurosciences, Royal Hallamshire Hospital, Sheffield Teaching Hospitals NHS Trust, Glossop Road, Sheffield, S10 2JF UK; 2 Academic Department of Neuroradiology, Royal Hallamshire Hospital, Sheffield Teaching Hospitals NHS Trust, Sheffield, UK

**Keywords:** Anti-GAD Ataxia, Gluten Ataxia, Gluten Free Diet, MR Spectroscopy, Immune Ataxia

## Abstract

The objective of this study is to report the clinical characteristics and treatment of patients with progressive cerebellar ataxia associated with anti-GAD antibodies. We performed a retrospective review of all patients with anti-GAD ataxia managed at the Sheffield Ataxia Centre over the last 25 years. We identified 50 patients (62% females) with anti-GAD ataxia. The prevalence was 2.5% amongst 2000 patients with progressive ataxia of various causes. Mean age at onset was 55 and mean duration 8 years. Gaze-evoked nystagmus was present in 26%, cerebellar dysarthria in 26%, limb ataxia in 44% and gait ataxia in 100%. Nine patients (18%) had severe, 12 (24%) moderate and 29 (58%) mild ataxia. Ninety percent of patients had a history of additional autoimmune diseases. Family history of autoimmune diseases was seen in 52%. Baseline MR spectroscopy of the vermis was abnormal at presentation in 72%. Thirty-five patients (70%) had serological evidence of gluten sensitivity. All 35 went on gluten-free diet (GFD). Eighteen (51%) improved, 13 (37%) stabilised, 3 have started the GFD too recently to draw conclusions and one deteriorated. Mycophenolate was used in 16 patients, 7 (44%) improved, 2 stabilised, 6 have started the medication too recently to draw conclusions and one did not tolerate the drug. There is considerable overlap between anti-GAD ataxia and gluten ataxia. For those patients with both, strict GFD alone can be an effective treatment. Patients with anti-GAD ataxia and no gluten sensitivity respond well to immunosuppression.

## Introduction

Glutamic acid decarboxylase (GAD) is the rate-limiting enzyme in the synthesis of the inhibitory neurotransmitter gamma-aminobutyric acid (GABA). GAD is found in both the central and peripheral nervous systems (including the enteric nervous system) as well as in pancreatic beta cells [1]. Antibodies against pancreatic islet cell proteins were first detected in children with insulin-dependent diabetes mellitus (IDDM) and were subsequently characterised as GAD antibodies [2–4]. Two major types of GAD enzyme exist, GAD65 and GAD67. These catalyse the formation of GABA at different cell locations and different time periods of development. The GAD67 enzyme is widespread in the central nervous system, whilst GAD65 is confined to nerve terminals. GABA is synthesised by GAD67 for neuronal activity unrelated to neurotransmission and synaptogenesis. On the other hand, GAD65 produces GABA for neurotransmission and is required at synapse [5].

The first neurological disease to be linked with GAD65 antibodies was stiff-person syndrome (SPS) [6]. GAD antibodies were subsequently shown to be present in the sera of up to 60% of patients with SPS, 80% of patients with IDDM (at a much lower titre than SPS), in patients with polyendocrine autoimmune syndromes and in some cases of sporadic, otherwise idiopathic ataxia [7–11].

The first case series of patients with so-called anti-GAD ataxia was published in 2001 [12]. It has since been common practice to include anti-GAD antibody testing in the diagnostic workup of all patients with progressive idiopathic ataxia. The presence of high titre of anti-GAD antibodies should alert the possibility of an immune-mediated ataxia and the need to consider immunosuppressive treatment.

We have previously demonstrated significant overlap between anti-GAD-associated neurological diseases and gluten sensitivity [13]. Here, we present our 25-year experience of managing 50 patients with anti-GAD ataxia at the Sheffield Ataxia Centre.

## Methods

This report is based on a retrospective observational case series of patients regularly attending the Sheffield Ataxia Centre. The South Yorkshire Research Ethics Committee has confirmed that no ethical approval is indicated given that all investigations/interventions were clinically indicated and did not form part of a research study.

## Patients

We performed a retrospective review of all patients with progressive ataxia and high serological titres of GAD antibodies (defined as > 2000 U/ml, normal < 5). Estimation of anti-GAD was made using a commercial assay (RSR Limited) according to the manufacturers’ instructions. Briefly, the wells are coated with GAD 65, and the samples are added and incubated. GAD65-biotin is added to the wells and incubated. Streptavidin-peroxidase is added to the wells. TMB substrate is added and incubated, and the plate is read at 405 nm (if low values, it is also read at 450 nm). Plate also includes calibrators 2000, 250, 120, 35, 18 and 5 U/mL and positive and negative control samples. The wells are washed in between each stage.

All patients have been seen, and most are still under active follow-up at the Sheffield Ataxia Centre, Sheffield, UK. All of these patients have been extensively investigated for other causes of ataxia including extensive genetic testing using next-generation sequencing [14]. The review included detailed examination of the clinical records and MR imaging including MR spectroscopy (MRS) of the cerebellum. This technique is under regular clinical use at our centre as a monitoring tool for all patients with progressive ataxia, particularly those undergoing therapeutic interventions [15]. In these series of 50 patients, we have only included patients presenting with cerebellar ataxia and excluded patients with SPS, the other group of patients seen in neurology clinics that often have high titres of anti-GAD antibodies.

## Results

### Clinical Characteristics

We identified 50 patients with anti-GAD ataxia (anti-GAD > 2000 U/ml, normal < 5). Sixty-two percent were female. The prevalence of anti-GAD ataxia amongst 2000 patients with progressive ataxia of various causes attending the Sheffield Ataxia Centre was 2.5%. Mean age at onset of the ataxia was 55 years (range 13–88) and mean duration 8 years (range 1–24). Gaze-evoked nystagmus was seen in 26%, cerebellar dysarthria in 26%, limb ataxia in 44% and gait ataxia in 100%. Using an established simple rating scale for severity of ataxia, 9 patients (18%) had severe ataxia (wheelchair bound), 12 (24%) moderate (needed walking aid) and the remainder 29 (58%) mild (could walk unaided) [16]. Twenty-four patients (48%) had autoimmune thyroid disease and 18 (36%) had IDDM. Other autoimmune diseases (excluding coeliac disease and gluten sensitivity) were seen in 13 patients (26%—6 pernicious anaemia, 3 Sjogren’s syndrome, 1 giant cell arteritis, 1 vitiligo, 1 Lambert-Eaton myasthenic syndrome, 1 myasthenia gravis). Only 5 patients (10%) had no evidence of additional autoimmune diseases. Family history of autoimmune diseases (first-degree relatives) was present in 52%. Thirty-five patients (70%) had serological evidence of gluten sensitivity, and 26 of these underwent gastroscopy and duodenal biopsy. Ten had coeliac disease on duodenal biopsy (villus atrophy, crypt hyperplasia and increased intraepithelial lymphocytes). The clinical characteristics of the complete cohort are summarised in Table 1.

**Table 1:** Clinical characteristics of 50 patients with anti-GAD ataxia

Total number of patients with anti-GAD ataxia	50 (62% female)
Mean age at onset of ataxia	55 years (range 13–88)
Mean duration of ataxia	8 years (range 1–24)
Gaze evoked nystagmus	26%
Cerebellar dysarthria	26%
Limb ataxia	44%
Gait ataxia	100%
Severe ataxia	18%
Moderate ataxia	24%
Mild ataxia	58%
Additional autoimmune diseases	90%
Family history of autoimmune diseases	52%
Serological evidence of gluten sensitivity	70%

### Imaging

Baseline MRI and MRS was available in 47/50 patients. Cerebellar vermian atrophy was present in 47% and cerebellar hemispheric atrophy in 36%. The atrophy of the vermis was graded as 1 (mild) in 15 of the patients, and the hemispheric atrophy was graded as 1 also in 15 patients. This meant that only 7 patients had either moderate or severe vermian atrophy with only 2 having moderate atrophy of the hemisphere. Progression of any atrophy over time was only observed in 2 patients.

The mean value of NAA/Cr ratio was reduced across the cohort at 0.892 (median 0.9, IQR 0.133). Thirty-four out of 47 (72%) patients had abnormal MRS of the vermis at the time of initiation of treatment, in keeping with what is often seen in immune-mediated ataxias [17].

### Neurophysiology

Neurophysiological assessments were done according to clinical indications. Twelve patients (24%) had clinical evidence of myoclonus which on EEG/EMG polygraphy recordings and quantitative analysis with jerk-locked averaging revealed a cortical generator of the myoclonus in three (2 had gluten sensitivity and one had IgA deficiency). The recording methodology and quantitative data analysis for the electrophysiological assessment of myoclonus is described in detail in previous publications [18]. Somatosensory evoked potential (SEP) recordings were also undertaken [19]. The possibility of long loop reflexes (C-reflexes) to look for stimulus sensitive myoclonus was examined with surface EMG electrodes in the leg and arm areas using published methodology [20]. Only one patient was found to have “giant” SEPs from the upper limbs but without any evidence of reflex myoclonus. Four patients were found to have large fibre axonal peripheral neuropathy on nerve conduction studies, one had evidence of LEMS (not suspected clinically) and one had typical features of SPS (continuous motor unit activity on needle EMG of paraspinal muscles) but without associated typical clinical features. One of the patients with electrophysiological evidence of cortical myoclonus also had on EMG polygraphy features of exaggerated auditory physiological startle responses elicited by unanticipated loud auditory stimuli at 100 dB. No habituation was seen despite the procedure was repeated on numerous occasions over short intervals of time, and consistently lower limb muscles were activated during the acoustic startle.

### Effect of Treatment

The 35 patients with serological evidence of gluten sensitivity (with and without coeliac disease) received detailed dietetic advice on strict adherence to a gluten-free diet (GFD). This was along the same lines as advice given for the treatment of gluten ataxia (GA) [21]. In 18 of the 35 (51%) patients with anti-GAD ataxia who also had gluten sensitivity, the ataxia improved on GFD alone, in 13 (37%) stabilised, and 3 patients have started the GFD too recently to draw conclusions. One patient continued to deteriorate despite GFD. Determination of clinical improvement/deterioration was based on clinical evaluation (repeated neurological examination during clinic attendances) and the patient’s own perception about their ataxia symptoms.

Mycophenolate was used in 16 patients (13 of which also had gluten sensitivity), 7 (44%) of which improved, 2 stabilised, 6 only recently started the medication and one did not tolerate mycophenolate. Only those patients with gluten sensitivity who stabilised but did not improve and the one who worsened despite GFD went on to be treated with mycophenolate.

At the time of starting treatment, the mean NAA/Cr ratio was 0.883 (median 0.9, IQR 0.115). Follow-up MRS after treatment was available in 20 patients to date with mean follow-up interval of 2.75 years (median 2 years and IQR 2.5 years). In keeping with clinical improvement, the follow-up MRS improved after treatment in 16 (14 on GFD alone and 2 on mycophenolate), worsened in 3 and was unchanged in 1. The mean NAA/Cr ratio after treatment for the 16 patients that improved increased to 0.954 (median 0.975, IQR 0.15) which was now within the normal range. Clinical improvement was observed in all 16 patients with increased NAA/Cr ratio on MRS. This was in keeping with what we have already published, that is, significant positive correlation between MRS and clinical improvement as assessed by the scale for assessment and rating of ataxia (SARA) [15, 22].

Three patients (not gluten sensitive) received intravenous immunoglobulins but with no evidence of sustained improvement. One patient received rituximab after no response to intravenous immunoglobulins, partial response to mycophenolate and cyclophosphamide (see illustrative clinical case). Another patient also received cyclophosphamide with good response. Only 9 of the 50 patients did not have any therapeutic intervention. This was either due to patient choice (6) or due to no clinical and MR spectroscopy evidence of disease progression (3).

Overall treatment with GFD and/or immunosuppression was associated with clinical improvement or stabilisation in 84% of those patients treated.

### Illustrative Clinical Case

**Fig. 1:**
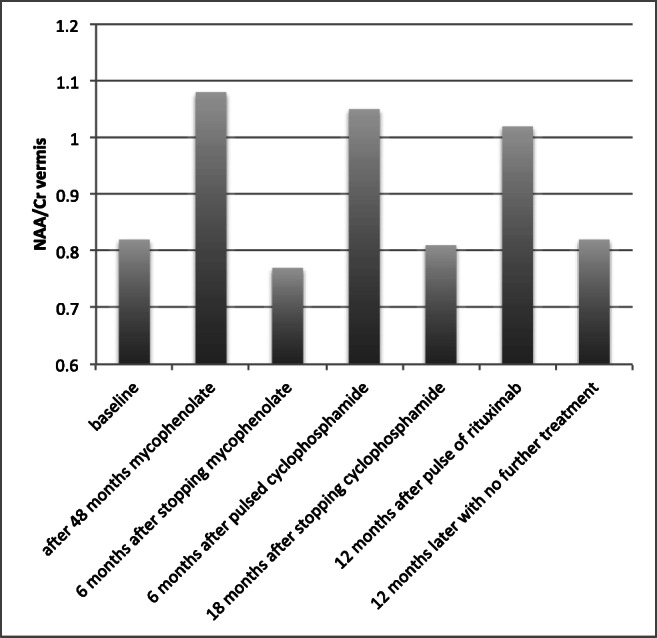
N-acetyl aspartate to creatine ratio (NAA/Cr) of the vermis showing alterations in relation to different treatment interventions. The patient developed ataxia in 1996, but the diagnosis of anti-GAD ataxia was not made until 2003. She followed a benign course initially. She did receive 3 courses of intravenous immunoglobulins in 2005 with no evidence of clinical benefit. Whilst there was evidence of significant improvement of the MR spectroscopy with subsequent interventions (from 2011 onwards, when MR spectroscopy became available), the patient did not feel that there was a dramatic clinical improvement of the ataxia and was reluctant to continue such treatments. She now has severe ataxia with disabling cerebellar tremor

**Fig. 2:**
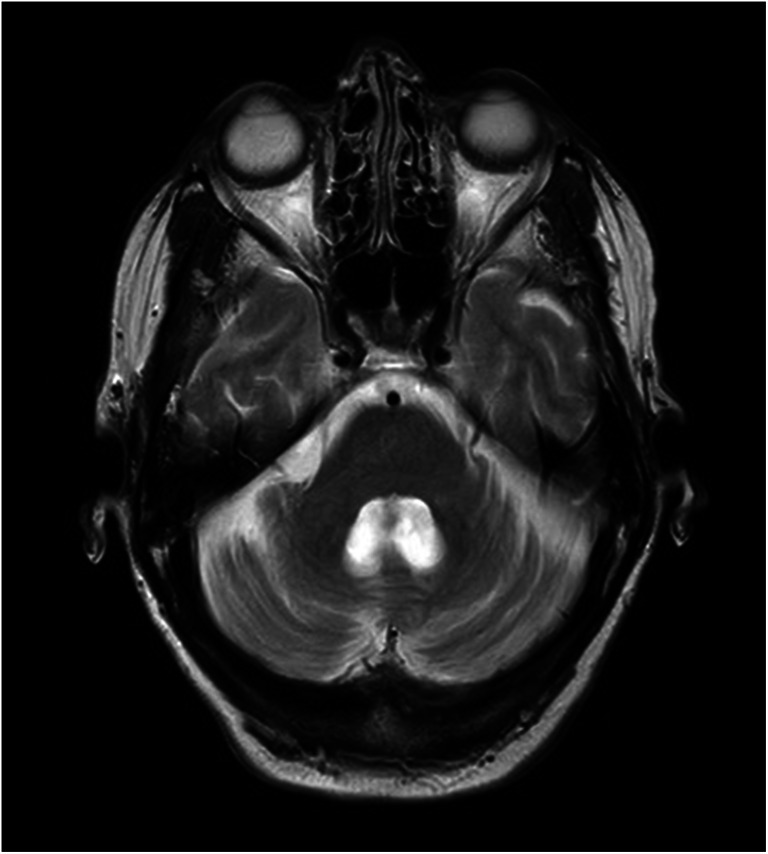
MRI scan from the illustrative case discussed in the text taken in 2020, which was 23 years after the original presentation. Whilst the baseline scan was normal, the scan above demonstrates significant atrophy of both the vermis and the hemisphere

A 50-year-old lady presented initially to audiovestibular medicine with a history of dizziness and vertigo in 1996. Investigations at the time did not identify any inner ear pathology; hence, she was referred to neurology for further evaluation. There was a past history of hypothyroidism and no family history of any neurological illness. She complained primarily of gait instability but was able to walk without walking aids and had no falls. Clinical examination showed some difficulties in tandem walking in keeping with gait ataxia but no other neurological dysfunction. Investigations at the time (including genetic testing) and MRI were normal. CSF examination showed normal protein, no cells and was negative for oligoclonal bands. She was monitored on a 6 monthly basis with little evidence of any progression. In 2002 at the age of 56 she developed IDDM and started insulin injections. In 2003 she was tested for the first time for anti-GAD antibodies and was positive with titres > 2000 U/ml. Despite the absence of any serological evidence of gluten sensitivity, she tried GFD for a year without any evidence of clinical improvement. Close monitoring with clinical evaluation of her ataxia showed that her balance was getting worse (although still able to walk using a stick) as a result of which in 2006 she had 2 courses of intravenous immunoglobulins without any evidence of improvement. In 2009 she was found to have low vitamin B12 (positive intrinsic factor) and started on B12 injections. After a period of stability, she again started deteriorating during 2011 when in addition to the progression of gait ataxia she complained of vertigo. Examination showed no evidence of vestibular pathology to account for the vertigo. In 2011 she underwent MR spectroscopy assessment of the cerebellum for the first time. In 2013 she started treatment with mycophenolate resulting in clinical stabilisation with MR spectroscopy evidence of improvement in NAA/Cr ratio of the vermis (Fig. 1). The patient, however, did not experience any clinical improvement, and 2 years later at her request, she stopped the mycophenolate. A few months later, there was a sudden deterioration that occurred during a summer holiday (2015). Investigations showed no concurrent infection on any other pathology to be contributing to the sudden deterioration. Repeat MR spectroscopy showed a significant drop of NAA/Cr from the vermis. She was treated with pulses of cyclophosphamide which again resulted in clinical stabilisation and improved MR spectroscopy. In early 2018, she received the first course of rituximab, and this was repeated later on in that year. This was again accompanied by MR spectroscopy improvement. Unfortunately by this stage, she had become significantly disabled due to cerebellar tremor that affected both arms and head. She had become largely wheelchair dependent. She decided not to continue with rituximab and is awaiting deep brain stimulation procedure to help with her disabling tremor. MR imaging at this stage (21 years after initial presentation) showed significant cerebellar atrophy (Fig. 2).

## Discussion

This report describes our series of patients with anti-GAD ataxia. Unlike previous smaller reports on the subject, all of the patients described here were clinically assessed and managed at the same centre, by the same team, being diagnosed after attending the Sheffield Ataxia Centre [23, 24]. A novel inference is the significant overlap of anti-GAD and GA, as 70% of patients with anti-GAD ataxia had serological evidence of gluten sensitivity and the majority responded to GFD alone without necessitating the use of immunosuppression.

There was a high prevalence of additional autoimmune diseases (90% of patients) and a family history of autoimmune diseases in first-degree relatives in 52%. This suggests that anti-GAD antibodies are a marker of multiple autoimmunity of which the cerebellar ataxia may be one of many autoimmune manifestations.

The association between anti-GAD antibodies, multiple autoimmunity and gluten sensitivity merits close consideration. Italian researchers have noted that the prevalence of additional autoimmune diseases in children with coeliac disease (CD) is significantly lower than in those patients with CD diagnosed in adulthood [25]. They concluded that GFD may reduce the risk of developing additional autoimmune diseases later on in life. This observation echoes our observed reduction in anti-GAD antibodies in patients with anti-GAD-related diseases and gluten sensitivity who go on a strict GFD [13].

Our results also highlight the preferential involvement of the cerebellar vermis, something that is commonly seen in immune-mediated ataxias [26]. The severity of anti-GAD ataxia by comparison to some degenerative (MSA-C) or genetic ataxias (SCA6) and some other immune-mediated ataxias (paraneoplastic cerebellar degeneration) seems, by comparison, mild. Indeed the majority (58%) had mild ataxia at presentation with only 18% having severe ataxia. The mild ataxia was also reflected in the MR spectroscopy measurements and the degree of cerebellar atrophy. However, if patients with anti-GAD ataxia remain untreated, their ataxia is generally progressive, with accumulation of significant disability over time. Treatment benefits most patients, but treatment should be considered at an early stage in an attempt to try and prevent the accrual of irreversible disability. The illustrative case reported here highlights this issue as the patient was not treated for 17 years, partly because the diagnosis of anti-GAD ataxia was made late. Her mild, slowly progressive ataxia seemed insufficient to justifying the use of immunosuppression for many years. This emphasises close clinical and MR spectroscopic monitoring, with a low threshold for treatment which is essential in preventing permanent disability. Of note is the slowly progressive course for years but with sudden and often severe irreversible exacerbations (as illustrated by the case report). This is another justification for early therapeutic intervention.

The pathological role of anti-GAD antibodies in the genesis of this immune ataxia is unclear. Since GAD65 is intracellularly located and is associated with a range of neurological conditions (ataxia, SPS, epilepsy) as well as IDDM, some have argued that anti-GAD65 antibodies have no pathogenic role to play. On the other hand, recent physiological studies in vitro and in vivo have demonstrated that binding of GAD65 by anti-GAD65 antibodies induces loss of GAD65 functions relating to GABA release, with an epitope dependence, leading to the development of cerebellar ataxia [27]. Given these observations, the question still remains as to why is GFD beneficial in those patients with gluten sensitivity and anti-GAD ataxia. The significant overlap between anti-GAD ataxia and gluten sensitivity also raises important considerations for anti-GAD pathogenicity. The response of anti-GAD ataxia to GFD in those patients who are gluten sensitive suggests that gluten sensitivity is part of the underlying pathogenesis. Furthermore, previous work from our group has highlighted a link between neurological manifestations of gluten sensitivity and anti-GAD antibodies, demonstrating reduction of the titre of anti-GAD antibodies after the introduction of GFD [13]. Indeed in 2 of the patients with anti-GAD ataxia and gluten sensitivity reported here, the level of anti-GAD dropped to below 2000 U/ml following GFD (unpublished observation). It is possible that such a drop may have been evident in other patients but the lack of routine quantification of high titres (> 2000 U/ml) by our clinical immunology laboratory does not allow such confirmation. In gluten ataxia, elimination of gliadin and transglutaminase (TG2 and TG6-related antibodies), all of which cross-react with cerebellar tissue, has a beneficial effect [28]. This would also likely be true in the context of patients with anti-GAD ataxia who are gluten sensitive. One possibility is that in these patients there is double insult causing cerebellar damage, one driven by gluten and the other by anti-GAD. Eliminating the first by strict GFD might help the ataxia (as observed here), but perhaps the combined use of both the GFD and immunosuppression may result in better overall outcome. In practice we have reserved the use of immunosuppression for those patients where GFD alone did not improve the ataxia. We have observed that patients with anti-GAD antibodies and gluten sensitivity proportionally are more likely to require additional immunosuppression than those patients with gluten ataxia alone even when both groups are on strict GFD. It is possible that a more effective approach would be to use both GFD and immunosuppression together at the time of diagnosis. The benefits of strict GFD can take up to a year to manifest and require complete elimination of all gluten-related antibodies that can only be achieved by strict adherence to GFD. Re-exposure to gluten causes reactivation of the immune process that results in cerebellar damage, and such dietary indiscretions may set the patient back for several months. These issues are challenging for many patients.

Although detailed neurophysiological assessments have not been done in all patients, there was electrophysiological evidence of cortical myoclonus in only three, two of which had serological evidence of gluten sensitivity and one had IgA deficiency (also suspected as having gluten sensitivity but negative serology due to the IgA deficiency). Gluten sensitivity is a well-recognised cause of cerebellar ataxia with cortical myoclonus and in this instance could have been a synergistic factor leading to the development of cortical myoclonus [18]. We have described a similar phenomenon in a cohort of patients with long-term exposure to lithium who were also gluten sensitive and developed cortical myoclonus [29]. In addition, our patient with the IgA deficiency was found to have evidence of exaggerated physiological acoustic startle responses. This tendency has been previously described in great detail in patients with SPS [30].

The presence of high titre of anti-GAD antibodies should not always preclude the investigation for other causes of ataxia. We have encountered 3 patients (not included in these series) with progressive ataxia, positive anti-GAD antibodies (> 2000 U/ml) but with alternative aetiology for their ataxia. The clinical suspicion of an alternative aetiology was as follows: One patient progressed rapidly and developed autonomic dysfunction fulfilling the criteria for probable MSA-C, a second patient in his 20s had severe global cerebellar atrophy (unusual for anti-GAD ataxia) which lead to genetic testing confirming a pathogenic mutation for ANO10 and a third patient had mild spastic paraparesis and waddling gait which lead to a genetic diagnosis of SPG7.

Whilst it is extremely rare to find such high levels of anti-GAD in genetic ataxias (in our genetic ataxia cohort, the figure for positive anti-GAD was 0.004%) in clinical scenarios where an alternative diagnosis is suspected examination of the CSF may prove helpful in demonstrating the intrathecal production of GAD antibodies thus further supporting the diagnosis of anti-GAD ataxia [12]. Indeed one may argue that the absence of a systematic study of the CSF is a limitation of this report. However, in everyday clinical practice, we found that high serological levels of anti-GAD in the context of an idiopathic ataxia were sufficient to diagnose and treat as anti-GAD ataxia.

Finally, we did not include in these series patients with SPS. In our experience, patients with SPS almost always have a degree of cerebellar ataxia, but their primary presentation is distinct and characterised by axial rigidity and spasms. SPS is also rare and in our experience less common than anti-GAD ataxia. Over the same period of time (25 years), we have encountered 18 patients with classic SPS (another area of our interest) as compared to 50 patients with anti-GAD ataxia. However, there may well be some ascertainment bias given that the patients with ataxia were seen at the Sheffield Ataxia Centre, one of only 2 National Ataxia Centres in the UK.

To conclude, we present here clinical data of a large series of patients with anti-GAD ataxia and highlight a significant overlap between anti-GAD and GA. This has significant therapeutic implications. We share our experience of treating these patients with GFD and/or immunosuppression and propose that early treatment intervention is more likely to be associated with better outcomes and limit permanent disability even if the initial presentation suggests a mild slowly progressive disease.

## Data Availability

Anonymised data can be shared after a reasonable request from any qualified investigator.
